# Integrated approaches to howler monkey (*Alouatta* spp.) medicine in professional care and conservation

**DOI:** 10.3389/fvets.2022.897404

**Published:** 2022-09-06

**Authors:** Enrique Yarto-Jaramillo, Irindi Çitaku, Carlos Enrique Rodríguez, Claudia Lewy Sánchez-Aldana, Mary Carmen Morales, Anneke Moresco

**Affiliations:** ^1^Instituto Mexicano de Fauna Silvestre y Animales de Compañía (IMFAC, S.C.), Mexico City, Mexico; ^2^Albanian Veterinary Association of Wild and Exotic Animals (AVAWEA), Tirana, Albania; ^3^Walt Disney Parks and Resorts, Orlando, FL, United States; ^4^Clinica Veterinaria Playa del Carmen, Playa del Carmen, Mexico; ^5^Centro Veterinario México, Mexico City, Mexico; ^6^Reproductive Health Surveillance Program, Grand Junction, CO, United States

**Keywords:** howler monkey, *Alouatta*, One Health, threatened species, One Conservation

## Abstract

Howler monkeys (*Alouatta* spp.) are threatened by anthropogenic pressures such as habitat fragmentation and deforestation, while conservation efforts are challenging to coordinate as natural geographic distribution ranges are the largest of any New World primate. On a One Health front, howler monkeys represent a great model to investigate the infectious disease dynamics between wild primates and humans as several infectious diseases affecting howlers have a demonstrated zoonotic potential. Howler monkey populations in professional care offer a window to investigate susceptibility to diseases in this species such as yellow fever (YF) and malaria, plus a myriad of endoparasite phyla, as well as vector-borne diseases such as Chagas disease and leishmaniasis. More studies are urgently needed to provide species-specific, medically relevant information as well as clinical descriptions of animals considered medically healthy. Moreover, howler monkeys are a challenging species to breed and maintain in professional care; additionally, reproductive parameters have been published only for a handful of species in this genus. On a One Health approach communication and collaborative health surveillance involving wildlife and zoo experts will ease the identification of factors that contribute to disease emergence facilitating the integration of human, animal, and environmental health. The One Welfare concept is based on the inextricable connection among animal welfare, human welfare, and environmental conservation. Integrating One Health and One Welfare into actions both *in-situ* and *ex-situ* will promote the sustainability of the forests and restoration of the ecosystems that those species inhabit, transitioning to a comprehensive One Conservation approach.

## Introduction

Howler monkeys (*Alouatta* spp.) are Neotropical primates in the family Atelidae and have the largest natural range of any New World Primate: from Mexico to northern Argentina (1, 2). Threats include anthropogenic pressures such as habitat fragmentation and deforestation, and in fact today more than 60% of *Alouatta* populations are found outside protected areas; therefore, conservation efforts are needed at the landscape level (3). Several anatomical and physiological features specific to howler monkeys put their populations at a higher risk: (a) a relatively large body size which makes them more prone to be hunted and also require more food, and potentially larger home ranges; (b) older age at sexual maturity and longer gestation time, which lowers their reproductive rate and lifetime reproductive output; (c) diurnal lifestyles and poor ability to live in open forests, which limits opportunities to forage and makes them more easily visible to some predators like humans (2).

From a One Health perspective, howler monkeys present a great model to investigate infectious diseases dynamics among and between wild primates, and humans thanks to their ability to survive in human-altered habitats, which brings them into regular contact with humans and domestic animals (3, 4). Assessing susceptibility to infectious and zoonotic diseases, gathering baseline clinical and reproductive data, and promoting focused research such as microbiome, nutrition, and overall population health assessments are some of the most important aspects of studying wild howler monkeys. Studying these aspects within inclusive frameworks and integrated actions connects the One Health and the One Conservation approaches and links *in-situ* and *ex-situ* conservation. Clinical investigations on *in-situ* and *ex- situ* populations should aim to standardize and validate among others blood and serum chemistry parameters, diagnostic tests for infectious and vector-borne diseases, radiographic and ultrasonographic anatomy, and establishing safe and effective anesthesia protocols. These are tools to ensure howler monkey welfare and successful studies of these animals *in-situ* and *ex-situ*. Standardized datasets of *ex-situ* populations can provide baselines to compare environmental changes, and in turn, the *in-situ* populations can provide normal ranges for the species. That is, standardized data allow us to be better stewards for the preservation of wildlife everywhere.

### Leveraging *ex-situ* populations

#### Collecting data from ex-situ populations to standardize tests and methods applicable and relevant to wild populations

Several infectious diseases that affect howlers have a demonstrated zoonotic potential, so identification of putative reservoir host(s) is one step in the investigation of these pathogens including pathogen evolution in reservoir wildlife populations, timely detection of outbreaks within identified sentinel species, reducing the risk at animal-human interfaces, and monitoring threats of zoonotic pathogens to native wildlife populations (2, 4). However, data collection of *in-situ* populations poses a number of challenges, making data collection from *ex-situ* populations an excellent alternative, as many diseases affect both populations (5, 6). Disease surveillance work can help detect new outbreaks and monitor the direction in which such outbreaks move, as well as host expansion, and also is a key aspect of managing *ex-situ* populations of *Alouatta* within a One Conservation framework (5, 7). Disease surveillance can occur through serology and molecular biology for specific diseases, coprology, necropsies, overall health evaluation with extensive clinical data collection, and in conjunction with ongoing behaviors studies which can be combined with data available for human populations (4–6, 8–14).

#### Validation of baseline clinical data in howler monkeys in professional care and its application to wild populations-the One Conservation approach

Individuals from threatened and endangered species kept in professional care are an excellent resource to generate baseline clinical information that can benefit their wild counterparts. Howler monkey populations in professional care offer a window to investigate disease susceptibility to various diseases and clinical problems in these species which can explain pathophysiologic processes that also affect wild populations. Most of the medical issues clinicians deal with in New World primate species in professional care are nutritional or infectious in nature (15). The literature regarding diseases in howler monkeys in professional care is sparse, so it is vital that clinical samples from individuals in zoological institutions are collected to standardize and validate data obtained from the numerous diagnostic methods available (16). The relatively controlled environment of a zoological institution, often well-known medical history of the animals, and documented husbandry practices present an opportunity for this validation to take place. At the time of the routine clinical examination, immediate processing of biological samples can take place [complete blood count (CBC), serum chemistry, urinalysis, coagulation tests, blood gasses, rapid infectious disease tests]. Cataloging radiographic and ultrasonographic anatomy during such exams presents an invaluable opportunity to collect and integrate information in a quick and efficient manner. Zoo-based diagnostic tests need to be validated for each species to provide species-specific, medically relevant information and clinical descriptions of clinically healthy animals, this allows for accurate interpretation of results. Standardizing these techniques and protocols under controlled conditions will facilitate performing these exams in the field, providing a framework to carry out these medical exams on wild populations. Zoological parks and collections with captive or semi-captive non-human primates such as howlers in high-risk areas of Chagas disease, are spots of high interest to study the potential transmission to humans and domestic animals. In Mexico alone, 19 of 31 triatomine species naturally infected with *T. cruzi* have been found in human houses, while 10 of 16 triatomine species infected with this protozoan have been found in the Amazon Basin (17).

#### Reproductive health as part of One Conservation

The One Health concept is usually associated with infectious disease or toxicology as they are the aspects that can most easily affect multiple species (5). However, when considering conservation, reproduction is a key aspect, as many reproductive diseases affect multiple species (18), which could be studied both in the wild and in professional care, making it a fundamental part of One Conservation (7). Reproductive parameters such as longevity, age at maturity, gestation length, presence or absence of a lactational anestrus, inter-birth interval, and reproductive rate at the population level are important factors that determine how fast a population can reverse a declining trend or recover from die-off events such as after an infectious disease outbreak. These factors are part of calculations of risk of extinction by the IUCN (19), but many of these parameters are currently not known for all howler monkey species. It is known though that the reproductive parameters listed above can be influenced by the nutritional and health status of females (20). Additionally, with deforestation, fragmentation, and other habitat changes, it is possible that these reproductive parameters may change if populations are pushed into areas of suboptimal food sources, or higher exposure to zoonotic diseases, accelerating the risk of extinction. Changes in reproductive parameters can only be documented if baseline values for each species are collected. This is usually accomplished through the maintenance of updated animal records in zoological and conservation-focused institutions using different management electronic systems. Population reproductive rates are positively affected by the percentage of females that are pregnant each year, and negatively affected by pregnancy loss. The latter, however, is very difficult to quantify in the wild. *Ex-situ* facilities can provide some insight into pregnancy loss occurrences by regularly evaluating reproductive parameters, such as birth records, fecal hormone monitoring and voluntary or opportunistic ultrasound examinations of the females; further pregnancy monitoring *via* hormones or ultrasound in *ex-situ* populations can provide comparative data for *in-situ* populations (21–24). If ultrasound examinations are performed regularly, valuable *in-utero* growth data can be obtained as well as information on reproductive disease that occurs naturally (24). So, knowledge gained from *ex-situ* populations can be applied to *in-situ* populations and vice versa. Fecal hormone analysis provides a great non-invasive technique, while single ultrasound examinations performed as part of field projects which already include capture and anesthesia of the population could provide data on the percentage of pregnant females. Some studies have published data on hormone monitoring of *Alouatta* spp. (21), but to the authors' knowledge no studies on ultrasonography of fetal development have been published in *Alouatta*. Additionally, *ex- situ* facilities are uniquely positioned to study topics like nutrition, microbiome, stress, contraception, and their effects on reproductive potential, and because some reproductive parameters have been published for *A. caraya, A. palliata* and *A. seniculus* (25–27) and few other species of howler monkeys, conservation efforts would benefit greatly from addressing those gaps.

### Leveraging *in-situ* populations

#### Necropsies and histopathologic evaluation, sample collection and preservation

Most mortality events reported in wild howler monkeys over the past two decades have been associated with outbreaks of YF and have occurred in several countries within the natural range of wild populations (from Argentina and Brazil to Nicaragua) (28–31). Natural disasters that impact food availability and maternal nutritional status, changes in social dynamics, and infanticide have been identified as significant causes of infant mortality (32). Opportunities for complete necropsy and histopathologic investigation in wild populations are rare and often associated with mortality events. Consequently, necropsies performed following a standardized protocol for sample collection and cryobanking as well as histopathology on individual cases in professional care can offer an insight into diseases affecting howler monkeys. Furthermore, elucidating disease and mortality etiologies in populations in professional care can provide useful information that can positively impact individual animals in rehabilitation centers prior to release back into their natural habitat (16), engaging the One Conservation perspective (7).

#### Data collection from *in-situ* populations highlighting specific examples

To eliminate or reduce the factors of decline of wild populations of animals it is of the utmost importance to understand the specific diseases affecting wildlife. For example, YF and malaria are important zoonotic diseases that affect wild *Alouatta* spp. (4, 28, 29) and thorough knowledge of disease ecology will facilitate appropriate One Conservation policies. Forest fragmentation and high humidity have a negative gradient for parasite richness and a positive gradient for levels of parasitism in howler monkeys. Potential exchange of parasites has been reported between humans and wild non-human primates in different regions of the world (4, 14, 29) and is an important factor for conservation. That is the investigation of endoparasites in howler monkeys grants us a glimpse into the ecological significance of these parasites and their impact on conservation and public health, highlighting the importance of the One Health approach (5). Several endoparasite phyla such as Apicomplexans, Ciliophora, Nematoda and Platyhelminths have been reported in wild howler monkeys in Ecuador, Mexico, Costa Rica, Belize, Argentina, and Brazil, but additional research is needed to further elucidate endoparasite impact on howler monkey populations and approximate the zoonotic spillover risk (6, 8, 9, 14, 33, 34). Natural infections of howler monkeys with Tripanosomatidae-*Trypanosoma cruzi* and *Leishmania* spp. have been identified in southeast Mexico. Serology for *Leishmania mexicana* shows that *Alouatta pigra* is more likely to develop leishmaniasis than *A. palliata* (35). Another example is Chagas disease, it is well-described in humans, non-human primates such as howler monkeys (*Alouatta caraya*) (36), and domestic dogs (17). *T. cruzi* has been found to affect twelve genera and over thirty species in New World primates, including howlers (17, 36).

#### Clinical data collection and research in *in-situ* primate populations

Challenges to wild primate health research are most relevant to ecological and behavioral aspects of wild primate populations. A collaborative approach between wildlife and zoo experts in health surveillance facilitates the identification of factors that contribute to disease emergence (37). Demonstrably, field work presents various challenges that complicate clinical research, among which we highlight: the need for capture of non-contained individuals, distance from sites with basic infrastructure such as electricity, running and drinking water, refrigeration, and freezing, limited communication and/or data and internet access, partial or total lack of diagnostic equipment, and insufficient personnel (38). Further, the interpretation and validation of field data represent one of the greatest challenges for the surveillance and research of wild animal diseases (37).

A pivotal component of current conservation integrated approaches is expanding knowledge on the ecology and transmission of diseases, which is hindered if the normal health parameters for that species are unknown. Therefore, this information must be collected through clinical and epidemiological-based studies in populations under professional care and replicated in wild animals. This may mitigate challenges faced by field studies and may help identify threats to wild populations and ways to remedy any negative impacts. Medical and diagnostic veterinary skills obtained working with the species *ex-situ* considerably facilitates application to *in-situ* populations. A great example of this is the long-term study and conservation of mountain gorillas (*Gorilla gorilla beringei*) in Africa with the One Health approach, incorporating clinical veterinarians whose basic and specialized training occurred in zoological collections handling great apes (39).

Baseline research on biochemical and hematological values in wild howler monkeys is needed to assess population health and the impact of anthropogenic activities, this has been done in Mexico, French Guiana, and Brazil (10–13). Similarly, possible lead poisoning due to anthropogenically driven habitat loss has been evaluated in Mexico (40) which deserves investigation in other countries in the natural range of howlers. Health data for this genus, along with information from *ex-situ* populations contributes to health assessments within the greater framework One Conservation.

### Conservation of wild howler monkeys—an integrated perspective

Several studies have proved the inextricable link between human and howler monkey health and disease in their natural range countries (2–4, 14, 29, 30, 33, 34) as well as the need of integration of the study of wildlife, human and domestic animal health. A modern all-inclusive wildlife medicine approach involving veterinarians, biologists, animal behavior experts, mathematicians, ecologists, experts in human health, and social and economic sciences collaborating for conservation (5, 13, 38) is urgent for the conservation of Neotropical non-human primates. This is how the concept of One Conservation is defined: integrating research in wild and in managed populations within the framework of a host of human and environmental factors (7).

The notion that *in-situ* conservation actions should be prioritized ahead of *ex-situ* management is outdated since both are equally important, furthermore the One Conservation concept proposes that they be conducted in an integrated way. Also, the argument that *ex-situ* conservation is expensive is short-sighted, as it disregards the fact that private corporations are extremely important players in conservation through zoos, aquaria, and scientific breeding centers, and invest in *ex-situ* actions. That is, while *in-situ* conservation strategies provide the best long-term options for biodiversity conservation, the short-term survival of many threatened species depends on *ex-situ* conservation strategies (41, 42).

Conservation of non-human primates in the Neotropics has been boosted by independent actions in several countries, including engagement of local communities to avoid activities resulting in howler monkey population decline such as hunting, illegal pet trade, and bushmeat (3, 4, 34, 42). These actions include the installation of bridges to reduce electric hazards, the installation of “ecological power lines” in critical areas for arboreal primates such as howlers (43), the relocation of groups/populations of howlers to power line-free areas, and reintroductions/translocations of howler monkeys from fragmented to protected habitats along with opportunistic biological and behavioral studies (unpub. data EYJ). Similarly, outreach focused on farming communities teaching the benefits of the presence and conservation of howler monkeys, education in rural settlements on infectious diseases potentially shared and diseases which are not shared between howler monkeys and other wildlife with local people and domestic animals has been instrumental for the protection of howler monkeys (4, 14, 29, 30, 34). Other initiatives such as the establishment of wildlife sanctuaries to support healthy howler monkey populations and other sympatric species released back into the wild have also encouraged specialists and International NGOs working together to build in-country capacity to accomplish a true One Conservation approach in the conservation of howler monkeys (44).

## Concluding remarks

Some researchers state that the One Health concept lacks the ecosystem component, but One Health aims to expand transdisciplinary collaborations and interactions among experts to enhance health care at the interface of humans, animals, and the environment. Habitat fragmentation, anthropic actions against wild populations such as hunting along with emerging infectious diseases account for some of the highest threats and challenges for the long-term survival of non-human primates across the globe. Some authors believe that it is essential to gather scientific knowledge on each species and their role in the landscape, and to integrate this knowledge into actions that promote the protection of the forests and ecosystems those species inhabit, thereby engaging the One Conservation approach.

One Conservation approach further promotes research into baseline values for the different species and populations of howler monkeys, which is critical to tailor the environmental policies to each geographical area. Focused disease surveillance through data collection in wild primates, including howler monkeys, warrants proactive sample collection and preservation. Investigation of reproductive health and parameters in human care can help understand the impact of various ecological changes and will facilitate *ex-situ* propagation of howlers.

One Conservation approach will serve to standardize protocols for *in-situ* and *ex-situ* investigations as well as the data and sample collection methodology including baseline bloodwork, coagulation parameters, infectious disease tests, radiography, and ultrasonography, among others. These data can be compared to data collected from populations in semi-wild and wild conditions and so identify potential areas to improve animal care and welfare under professional care and provide robust, science-based data to promote conservation of howler monkeys at a landscape level.

## Data availability statement

The original contributions presented in the study are included in the article/supplementary material, further inquiries can be directed to the corresponding author.

## Author contributions

All authors listed have made a substantial, direct, and intellectual contribution to the work and approved it for publication.

## Conflict of interest

Author CR was employed by Walt Disney Parks and Resorts. The remaining authors declare that the research was conducted in the absence of any commercial or financial relationships that could be construed as a potential conflict of interest.

## Publisher's note

All claims expressed in this article are solely those of the authors and do not necessarily represent those of their affiliated organizations, or those of the publisher, the editors and the reviewers. Any product that may be evaluated in this article, or claim that may be made by its manufacturer, is not guaranteed or endorsed by the publisher.
